# Preventing Alcohol-Related Harm in East Africa: Stakeholder Perceptions of Readiness across Five Countries

**DOI:** 10.3390/ijerph192214979

**Published:** 2022-11-14

**Authors:** Monica H. Swahn, Zakaria Robow, Adelaide Balenger, Catherine A. Staton, Rogers Kasirye, Joel M. Francis, Sophia Komba, Patterson Siema

**Affiliations:** 1 Wellstar College of Health and Human Services, Kennesaw State University, Kennesaw, GA 30144, USA; 2 School of Public Health, Georgia State University, Atlanta, GA 30302, USA; 3 Department of Emergency Medicine, Duke Global Health Institute, Duke University, Durham, NC 27710, USA; 4 Uganda Youth Development Link, Kampala P.O. Box 12659, Uganda; 5 Department of Family Medicine and Primary Care, University of the Witwatersrand, Johannesburg 2193, South Africa; 6 East Africa Alcohol Policy Alliance, Dar es Salam, Tanzania; 7 African Population and Health Research Center, Nairobi 00100, Kenya

**Keywords:** alcohol prevention, alcohol harm, research, capacity, stakeholder, academic-community partnerships, East Africa

## Abstract

Objective: While alcohol-related harm is a recognized public health priority, the capacity to address and mitigate its harm is lacking, primarily in low-income countries. Recent developments including new tools that can assess readiness for preventing alcohol-related harm, specifically in low-resource settings, can be used to determine strengths and opportunities for supporting, planning, and resource allocation. In this study, we determined the perceptions of readiness and capacity for the prevention of alcohol-related harm across East Africa among stakeholders engaged in such work. Methods: We conducted a cross-sectional survey in 2020, distributed by the East Africa Alcohol Policy Alliance to their member alliances and stakeholders across five countries in East Africa (i.e., Burundi, Kenya, Rwanda, Tanzania, and Uganda). The survey included modified measures from the Readiness Assessment for the Prevention of Child Maltreatment (RAP-CM) short form, organizational size and funding, research capacity and priorities, and perceptions related to alcohol prevention and harm both locally and in the region. Analyses were computed based on 142 persons/organizations completing the survey. Results: In terms of general readiness, the overall adjusted aggregate score for East Africa was 39.70% (ranging from 30.5% in Burundi to 47.0% in Kenya). Of the 10 domains assessed (on a 0–10 scale), across all countries, knowledge of alcohol prevention (8.43), institutional links and resources (6.15) and legislation, mandates and policies (5.46) received the highest scores. In contrast, measures pertaining to resources (i.e., material, human, technical, and informal) received the lowest score. Conclusions: Our results demonstrate substantial variability in the readiness to address alcohol-related harm across East Africa. The highest capacity was noted for knowledge towards alcohol prevention, institutional links, and legislative mandates and policies. However, important gaps were noted in terms of attitudes towards alcohol prevention, the will to address the problem, as well as material, human, and informal resources, which need to be urgently addressed to strengthen capacity for addressing and mitigating the significant toll of alcohol-related harm in the region.

## 1. Introduction

Alcohol-related harm represents an urgent global health concern that has not received adequate attention. Moreover, there is a disproportionate burden of alcohol-related harm in Africa, which is the WHO region with the highest alcohol-attributable burden of disease and injury [1]. Recent calls have been made to address the emerging risk factors that are associated with alcohol use and for African governments to be more proactive [2]. A recent study also highlighted that: “*Sub-Saharan Africa has long been characterized as a region with weak alcohol policies, high proportion of abstainers and heavy episodic drinkers (among drinkers) and as a target for market expansion by global alcohol producers*” [3].

The acknowledgment by leading alcohol prevention experts that there has been limited progress in addressing alcohol-related harm, further underscores the need to escalate global public health prevention and intervention strategies to mitigate alcohol-related harm [4,5]. The urgency of mitigating alcohol-related harm also stems from the pervasive use of alcohol and its contribution to both morbidity and mortality. Alcohol remains one of the most used substances world-wide and is a significant contributor to deaths (5%) and also the global disease burden (5%) [1]. What is perhaps even more troubling, despite this recognition of the large and disproportionate alcohol burden, research related to alcohol use and harm and evaluated alcohol interventions for individuals remain scarce across sub-Saharan Africa [6]. While there may be many underlying reasons for the limited alcohol research, the key factors are likely a lack of capacity, prioritization, and interest for this type of research [7,8,9].

Within Africa, there are important regional variations in levels of alcohol-related harm. While West Africa had the highest disability-adjusted life years (DALYs) lost in 2016 due to alcohol, East Africa followed as the African region with the second highest number of DALYs [3]. The region is also a focus for alcohol marketing and market expansion of various alcohol products [3], which will further increase alcohol use and related harm. Together, these concerns collectively indicate the need to address alcohol use and related harm in both West and East Africa. However, regional data are rarely available or presented, and alcohol research remains relatively scarce when factoring in the overall burden and levels of alcohol-related harm in these regions.

While research remains lacking, East Africa has seen some important changes over the past 10–15 years with respect to alcohol policy development and control measures. For example, Kenya implemented the Alcohol Drinks Control Act in 2010, also known as the Mututho law. The Mututho law is often referred to as a model policy for other countries in the region [10]. It is important to recognize, however, that this act is not considered a national policy, as county governments chose to ratify the act separately from the government’s devolution [10]. A review of alcohol policies across Africa finds great variation in terms of strength and policy restrictiveness [11]. New developments include the Alcohol Control Policy in Uganda, which was adopted in 2019 by the Cabinet [12]. Similarly, sachet-packaged local gin, a form of mostly unrecorded alcohol, was banned in Uganda in 2019 [3]. However, it is also noted that there is insufficient enforcement [3] and a limited priority setting for research [7].

It is within this context of fragmented infrastructure, limited alcohol policies and laws, that some countries largely abdicate their responsibilities and instead engage community-based organizations (CBOs) and non-governmental organizations (NGOs) to steer prevention initiatives and strategies for preventing alcohol-related harm. In low-resource settings, as in East Africa, CBOs and NGOs are unlikely to have adequate resources, capacity, or tools to mitigate the burden of alcohol-related harm. However, their capacity to prevent alcohol-related harm has not been systematically assessed, if at all. In fact, few tools have been used in research to assess the strengths and weaknesses of stakeholders for planning and resource allocation purposes [9]. This lack of applicable tools and resources has likely served as a critical obstacle for research, intervention, and policy development in both West and East Africa.

In a previous and recent study [9], we adapted the WHO instrument, the Readiness Assessment for the Prevention of Child Maltreatment (RAP-CM) [13], for another use. The instrument modification allowed us to conduct a Readiness Assessment for the Prevention of Alcohol-Related Harm (RAP-ARH) in low-resource settings. The RAP-CM is a well-recognized tool which has been successfully and previously used in several regions and countries (e.g., the Middle East, Brazil, Macedonia, Malaysia, Kenya, and South Africa) [14,15,16,17,18,19]. This tool has ten dimensions which include stakeholders’ attitudes, perceptions, and knowledge of Child Maltreatment (CM), availability of data on CM, willingness to take action and address CM, and the legal, policy, human, and technical resources available to prevent CM [13].

We modified the tool and implemented it to use stakeholder input from NGOs and CBOs across West Africa [9] to determine readiness to address alcohol-related harm. The original tool, the RAP-CM, was developed to identify key gaps in capacity or readiness and to provide a baseline for tracking progress, to guide resources, identify interventions, to spur action, and be used as a teaching tool for stakeholders [13], and that is also how we used it, but instead to assess alcohol-related harm. The adaptation and implementation of this tool worked very well across West Africa, with positive feedback from stakeholders and the engaged alcohol alliances who found the information valuable in their planning, training, and advocacy efforts. As such, we also implemented it across East Africa, another priority region for mitigating alcohol-related harm and in urgent need of capacity strengthening. The research objective of this study was to determine the readiness and capacity for preventing alcohol-related harm across five countries in East Africa.

## 2. Materials and Methods

### 2.1. Design

Our study design included a brief cross-sectional online survey administered to stakeholders engaged in alcohol prevention, outreach, and policy development in collaboration with the East Africa Alcohol Policy Alliance from October to December 2020. The project was named the “East African Alcohol Policy Alliance Capacity Assessment Survey (EAAPACAS), and followed the same procedure described elsewhere for the same survey conducted in West Africa [7,8,9]. The research objective of the online survey was to assess the organizational structure, operational and strategic priorities, research capacity, target population of services provided, perceptions of best practices, and alcohol-related concerns in their local communities, and familiarity with the WHO SAFER initiative [20]. Within the longer survey, we also included a specific readiness assessment tool referred to as the RAP-ARH, which is the focus of this article.

### 2.2. Participants

In terms of participant recruitment, we used a snowball strategy where survey invitations were distributed to those affiliated with EAAPA via email and on social media platforms (i.e., WhatsApp and Facebook) to complete the anonymous Qualtrics online survey. The survey targeted respondents, predominantly from NGOs and CBOs, in five East African countries. Participants did not receive any compensation for taking the survey and participants were free to invite others. Participants represented all five selected countries (i.e., Burundi (n = 14), Kenya (n = 42), Rwanda (n = 9), Tanzania (n = 36), and Uganda (n = 41)) and most participants worked at or represented non-governmental or community-based organizations. Because of the survey distribution approach, a response rate could be computed. The survey was deemed exempt and approved by the Georgia State University Institutional Review Board (H21183).

### 2.3. Instrument

Briefly, within the longer EAAPACAS, we included a modified version of the RAP-CM [13]. The research team closely reviewed all survey questions in the RAP-CM and replaced any term reflecting child maltreatment with “alcohol-related harm” and made minor editorial changes as needed to create the Readiness Assessment for the Prevention of Alcohol-Related Harm (RAP-ARH). We also added a definition (as was done in the original tool) for alcohol-related harm, as follows: “*For this project, we define alcohol-related harm broadly to include any potential alcohol-related harm to the drinkers as well as harm to others. Alcohol harm may include biological concerns such as HIV transmission, liver damage or cancers, psychological and societal harms including addiction/alcohol use disorders, physical and psychological abuse and violence, injuries, car crashes and other types of harm*”.

Additionally, we made a few modifications to the formatting of the response options to facilitate the online survey distribution. The original RAP-CM short form tool is comprised of 19 survey questions; 14 of which are presented with categorical response options, 2 with open-ended “write-in” responses, and 3 where participants are encouraged to write in and list names of programs, names of institutions, and specific partnerships. To simplify the online survey distribution, we modified the two open-ended survey questions where participants were asked to list the consequences of child maltreatment and also the risk factors for child maltreatment. Rather than having participants create their own list, we provided a list of risk factors and outcomes for alcohol-related harm so that participants could select all options they thought were applicable. Each list comprised 10 items and an option for “other”, where participants could write their own response. Please refer to the previous published report regarding the specific and detailed survey modification and adjustments for the RAP-ARH [9].

### 2.4. Scoring

The method used to compute the Readiness Assessment RAP-ARH scoring closely followed the RAP-CM short version scoring system [21]. Again, this scoring measure was intended to indicate a country’s or region’s preparedness to adopt alcohol prevention and policy programs. As used with the RAP-CM scoring, our approach was divided into a 10-dimension model: (1) attitudes toward alcohol-related harm prevention; (2) knowledge of alcohol-related harm prevention; (3) scientific data on alcohol-related harm prevention; (4) current programs and evaluation; (5) legislation, mandates, and policies; (6) will to address the problem; (7) institutional links and resources; (8) material resources; (9) human and technical resources; and (10) informal social resources (non-institutional) [13].

### 2.5. Data Analysis

The RAP-CM short version scoring system was used to assess both aggregate and country-specific scores [21]. Responses to the survey questions were used to produce a score for each of the 10 dimensions (ranging from 0–10). The score was determined by taking the frequency value from the survey output multiplied by 2, 1, or 0 as identified using the RAP-CM short version scoring guidelines. For any dimensions that required two-part scoring, a weighted average was taken to receive a score. Additionally, as outlined in the scoring guidelines, we also used the 2.5 multiplier, which gives each dimension a score ranging between 1 to 10. To calculate the overall aggregated readiness score (%), we summed the individual dimensions scores, creating a possible score ranging between 0–100. The dimension scores were mapped in Microsoft Excel in the form of a radar chart. The analyses for this report were based on an analytic sample of n = 142, as we omitted participants who did not specify the country where they work and/or with missing data on the scoring of the RAP-ARH.

## 3. Results

Overall, among the 142 participants included in the analytic sample, organizations represented NGOs (56%), CBOs (17%), governmental organizations (8%), universities (6%), international organizations (1%), research institutes (1%), and other/unspecified (11%). The countries that were represented among survey respondents included Kenya (30%), Uganda (29%), Tanzania (25%), Burundi (10%), and Rwanda (6%).

**Figure 1:**
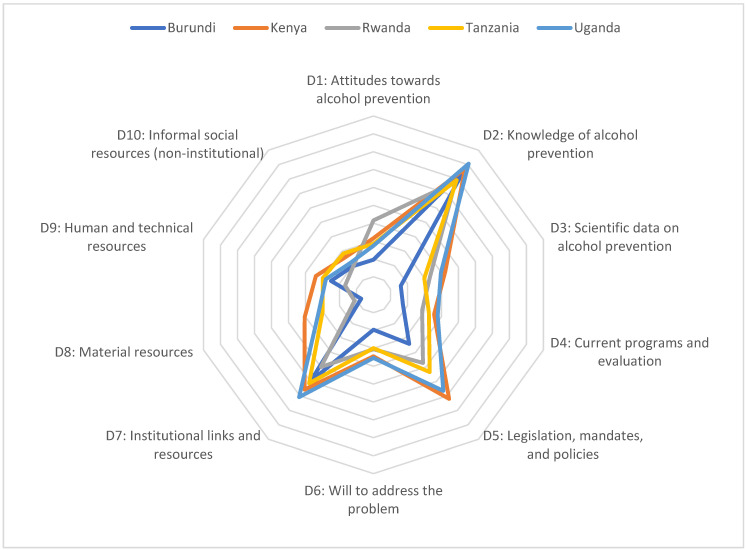
Mean dimension scores from the readiness assessment for prevention of alcohol-related harm using a 10-Point Scale across ten domains (D1–D10) in five countries (EAAPACAS; n = 142).

The overall readiness score for East Africa was 39.7% (ranging from 30.5% in Burundi to 47% in Kenya). The scores for each country and each domain are presented as radar charts (Figure 1) and in a table format (Table 1). Of the 10 dimensions (D1–D10), ranging in scores from 0–10, the highest score in this region pertained to D2: Knowledge of alcohol prevention (8.4); D7: Institutional links and resources (6.15) and D5: Legislation, mandates and policies (5.5). However, there were substantial variations across countries, specifically for D5: Legislation, mandates and policies, where Burundi and Rwanda had lower scores. With respect to legislation, mandates, and policies, 57% of participants across East Africa indicated yes to governmental and non-governmental agencies officially mandated to address alcohol-related harm. However, with respect to whether an official policy exists or is in place that specifically addresses alcohol-related harm, only 47.9% of participants said yes.

In contrast, the lowest domain scores were observed for D10: Informal Social Resources (2.32); D8: Material Resources (2.42); and D9: Human and Technical Resources (2.67) (Figure 1). Regarding Informal Social Resources attitudes toward alcohol prevention, more than half of participants (63.4%) indicated that citizen participation to address health and societal problems in the country is low or moderate (Table 2). Moreover, more than half of participants (57.0%) indicated that people in the country are moderate or poor at getting things done. The factor with the most variation across countries pertained to material resources, where Burundi had the lowest score (0.72) and Kenya had the highest score (4.05) (Table 2).

## 4. Discussion

The objective of this study was to determine readiness for the prevention of alcohol-related harm across East Africa. We implemented a modified tool used originally by the WHO to assess readiness for the prevention of CM (RAP-CM) in low resource settings [13]. We followed the same approach we had previously used in West Africa [9]. Our findings showed high perceived knowledge of alcohol-related harm, strong legislative mandates and policies, and institutional links and resources across East Africa. However, substantial variations between countries were noted. Overall, the readiness score for East Africa was 39.70% which is lower than that observed across West Africa of 45.0% [9].

With respect to the strengths noted in this readiness assessment, they were very similar to the findings previously noted in West Africa, in particular with respect to perceived knowledge of alcohol-related harm, and also strong legislative mandates and policies [9]. However, unlike the results observed in West Africa, in East Africa, the institutional links and resources scored much higher and may perhaps explain some of the recent progress seen in that region with respect to both policy development and research. In contrast, in West Africa, institution links and resources were outlined as a key limitation. Perhaps, even within the context of limited resources in all its forms, those institutional links and social resources may make a difference in terms of collaboration, leveraging expertise and institutional capacity. As has been previously noted, there is limited research output from West Africa, which may be partially due to weak institutional links, limited data, and limited human and technical resources [22,23,24].

Our findings noted areas that need urgent attention. In particular, we noted that resources, whether material, human, or technical, and informal resources were the lowest ranked of all dimensions examined. However, the remaining four indicators also ranked low, reflecting low support or attitudes toward alcohol prevention, limited data, limited programs and evaluation, and limited willingness to address the problem which, taken together, present a broad need for capacity building and resources to mitigate alcohol-related harm across East Africa.

These findings present a grim outlook for stakeholders engaged in preventing alcohol-related harm, as there are significant hurdles to progress across East Africa as resources appear scarcer and more competitive. Unfortunately, there are few funders for international alcohol prevention work, and many of those funders have other competing priorities. As such, it is unclear what the future may hold in terms of resource allocation and capacity building for those working in alcohol prevention in East Africa. However, assessments like these can illustrate key needs and strengths, and leverage those to maximize impact. Findings from this and similar studies [9] can encourage researchers in settings with more resources to lend their expertise, participate in tool development, and commit to capacity-building strategies to mitigate alcohol-related harm. There are several recent examples that can be leveraged and used in low-resource settings to strengthen capacity where it is urgently needed [7,8]. Moreover, there is a clear need for investments at both the national and local levels. Even when policies are in place, they are often not supported with any or adequate resources.

It is clear that alcohol-related harm represents a grave disparity, mostly impacting countries with limited resources and capacity to fully address the issue, in part because of strong alcohol industry influences from multinational companies owned by entities outside of Africa [9]. These external and undue influences limit policy development and enforcement, drain resources, and are further exacerbated by aggressive alcohol marketing [9]. Recent research indicates that alcohol marketing is strongly associated with heavy and problem drinking among youth in Kampala [25], adding to extensive research in other parts of the world that alcohol marketing causes underage drinking [26]. As such, a clear strategy for reducing alcohol-related harm, at least among youth, is to restrict alcohol advertisements, a recommended strategy by the WHO. However, in the absence of more data, despite the will to support prevention and the resources and infrastructure to mitigate alcohol-related harm, no tangible progress can be made or expected. Meanwhile, our adapted tool identifies key strengths and limitations that can be considered for priority settings and planning, or for evaluating progress across a country or region.

However, there are important limitations of this research that should be considered when interpreting our findings. First, the analytic sample of respondents (n = 142) may limit the generalizability of the results to other settings. This is perhaps most relevant to the findings stratified by country. The East Africa Alcohol Policy Alliance and partners invited potential participants among their networks of engaged alliances and stakeholders. Accordingly, those organizations not affiliated with the alcohol policy alliances may not have been invited to participate. Additionally, we deliberately did not seek participants from governments or universities. These recruitment strategies may further limit the generalizability of the findings to broader stakeholders and settings. Finally, because of the study design used, it was not possible to compute response rates by country or for the overall sample.

Even so, and in spite of these limitations, these findings represent the first attempt to identify key themes, strengths, and limitations in the field of alcohol-related harm reduction in East Africa. Moreover, this assessment was previously implemented in West Africa and used to brief stakeholders and decision makers. As such, it has already been helpful to those engaged in this work. However, it is important to note that this assessment was not designed to infer precision in the findings in the region or for a specific country. It was adapted and implemented with the goal of identifying broader issues for further discussion in order to strengthen readiness for the prevention of alcohol-related harm. Additionally, while it can be used to provide a capacity assessment at one point in time, it can also be used to assess changes in preparedness or capacity over time. The tool is easy to use and score, and can be very helpful to compare findings across countries, settings, or communities, and can even be delivered online as it was for this study in East Africa and also in West Africa [9]. It can also be used as an evaluation tool following capacity-building initiatives.

To our knowledge, the RAP-ARH is the first and only tool that has been used to specifically assess readiness and capacity for preventing alcohol-related harm across several key domains, in a low-resource setting. A previous and important related tool, the National Alcohol Policy Score Card (NAPSC), had 12 measurements and was implemented in South Africa in 2006 and 2011 to determine the state of alcohol policy development and its implementation [27], but it has not been implemented since in any other country, to our knowledge. The development of new tools for research, evaluation, and capacity building is critically important for progress in preventing alcohol-related harm.

## 5. Conclusions

In this study, we found that the modified tool (RAP-ARH) has been very helpful in assessing the domains that are in urgent need of attention for progress to be made in terms of mitigating alcohol-related harm in East Africa. This remains a relatively understudied region of the world that sadly bears a very high burden of alcohol-related harm [3]. The approach we have used can easily be replicated and the findings easily conveyed for broader stakeholder input and planning, as well as additional research. There are clear priorities for next steps and capacity building that can be discussed with stakeholders to address the urgent resource needs and attitudes toward alcohol prevention, the will to address the problem, and current programs and evaluations; all of which represent areas in need of strengthening across East Africa.

## Figures and Tables

**Table 1:** Mean Dimension Scores from the Readiness Assessment for Prevention of Alcohol-Related Harm using a 10-Point Scale across Ten Domains (D1–D10) in Five Countries (EAAPACAS; n = 142).

	Burundi	Kenya	Rwanda	Tanzania	Uganda	East Africa
D1: Attitudes towards alcohol prevention	1.96	3.16	4.16	2.85	2.74	2.97
D2: Knowledge of alcohol prevention	8.65	8.72	7.81	7.92	9.06	8.43
D3: Scientific data on alcohol prevention	1.61	4.23	3.33	2.99	3.97	3.23
D4: Current programs and evaluation	1.73	3.57	2.86	3.26	3.78	3.04
D5: Legislation, mandates, and policies	3.4	7.2	4.72	5.35	6.65	5.46
D6: Will to address the problem	1.96	3.45	3.05	2.99	3.54	3.0
D7: Institutional links and resources	6	6.56	5	6.13	7.08	6.15
D8: Material resources	0.72	4.05	1.11	2.99	3.23	2.42
D9: Human and technical resources	2.5	3.39	1.67	2.99	2.8	2.67
D10: Informal social resources (non-institutional)	1.97	2.62	1.94	2.85	2.2	2.32
Overall Adjusted Aggregate Score %:	30.5	47.0	35.7	40.32	45.05	39.70

**Table 2:** Responses to Readiness and Capacity Assessment for Prevention of Alcohol-related Harm in East Africa and by Country (EAAPACAS; n = 142).

	East Africa (%) **	Burundi (%)	Kenya (%)	Rwanda (%)	Tanzania (%)	Uganda (%)
Dimension 1: Attitudes towards alcohol-related harm						
How much of a priority is alcohol-related harm prevention?						
High Priority	18.30	7.10	26.20	22.20	16.70	14.60
Moderate Priority	26.80	14.30	28.60	44.40	27.80	24.40
Low Priority	47.20	64.30	33.30	22.20	50.00	58.50
Do you think that measures taken so far to prevent alcohol-related harm in your country have been adequate?						
Adequate	6.30	0.00	4.80	22.20	8.30	4.90
Neither adequate nor inadequate	40.10	50.00	35.70	33.30	36.10	46.30
Inadequate	47.90	42.90	47.60	33.30	55.60	46.30
Dimension 3: Scientific data on prevention of alcohol-related harm						
Are there data on the magnitude and distribution of alcohol use in your country? If so, how good is the quality of these data?						
Yes, limited data exist, and their quality is good	15.5	0.00	16.70	22.20	8.30	24.40
Yes, such data exist, and their quality is good	7.0	0.00	9.50	11.10	8.30	4.90
Yes, such data exist, but their quality is low or fair or you do not know the quality	29.60	28.60	38.10	11.10	30.60	24.40
No	12.70	21.40	9.50	0.00	16.7	12.20
Are there data on the magnitude and distribution of alcohol-related harm in your country? If so, how good is the quality of these data?						
Yes, limited data exist and their quality is good	18.30	0.00	26.20	22.20	11.10	22.00
Yes, such data exist and their quality is good	4.90	0.00	4.80	11.10	2.80	7.30
Yes, such data exist, but their quality is low or fair or you do not know the quality	31.70	35.70	31.00	11.10	38.90	29.30
No	17.60	28.60	16.70	0.00	19.40	17.10
Dimension 5: Legislation, mandates, and policies						
Are there any governmental or non governmental agencies officially mandated to address alcohol-related harm prevention in your country?						
Yes	57.00	14.30	71.40	44.40	50.00	65.90
No	10.60	28.60	4.80	22.20	13.90	4.90
Is there an official policy, or are there official policies, specifically addressing alcohol-related harm in your country?						
Yes	47.90	14.30	69.00	22.20	33.30	56.10
No	21.10	50.00	2.40	33.30	33.30	17.10
Dimension 6: Will to address the problem						
Are there political leaders who express strong commitment to the issue of alcohol-related harm and are taking effective measures to address the problem?						
Yes	28.20	7.10	26.20	44.40	25.00	36.60
Not Clear	38.80	35.70	35.70	11.10	36.10	34.10
No	19.00	35.70	19.00	11.10	16.70	17.10
How intensive have communication efforts been concerning alcohol-related harm in your country?						
Intensive	2.10	0.00	2.40	0.00	0.00	4.90
Moderate	33.10	28.60	45.20	22.20	33.30	24.40
Weak	35.90	35.70	26.20	33.30	38.90	43.90
Dimension 8: Material Resources						
Does the Ministry of Health (or equivalent) in your country have a dedicated budget for alcohol-related harm prevention?						
Yes	12.00	0.00	9.50	11.10	11.10	19.50
No	31.70	14.30	40.50	11.10	33.30	31.70
Are there dedicated budgets in other parts of government (e.g., other ministries, departments, etc.) in your country?						
Yes	19.00	0.00	42.90	0.00	13.90	9.80
No	27.50	14.30	16.70	11.10	36.10	39.00
Dimension 9: Human and Technical Resources						
Do you think the number of professionals specializing in alcohol-related harm is adequate for large-scale implementation of alcohol-related harm prevention						
Yes	7.70	7.10	9.50	0.00	13.90	2.40
No	58.50	57.10	59.50	44.40	52.80	65.90
How widely available are undergraduate or postgraduate educational institutions which devote some of the curriculum to alcohol related harm prevention?						
Widely available	2.10	0.00	0.00	0.00	5.60	2.40
Some or a few	38.70	28.60	57.10	22.20	27.80	36.60
None	23.20	28.60	9.50	22.20	33.30	26.80
Dimension 10: Informal Social Resources						
What level of citizen participation is there typically in efforts to address various health and social problems in your country?						
High	4.20	0.00	9.50	0.00	2.80	2.40
Moderate	33.80	28.60	28.60	44.40	44.40	29.30
Low	29.60	28.60	31.00	22.20	22.20	36.60
How good at getting things done through their joint efforts are the people living in your country?						
Good	11.30	14.30	16.70	0.00	13.90	4.90
Moderate	33.10	21.40	23.80	33.30	36.10	43.90
Poor	23.90	21.40	31.00	22.20	19.40	22.00

Note that Dimensions 2, 4, and 7 were not asked as categorical response questions and are not presented in this table. ** Overall numbers represent all participants including those with countries with small cell sizes not presented in the table.

## Data Availability

Data is available from the lead author upon request.

## References

[1] World Health Organization (2018). Global Status Report on Alcohol and Health. https://apps.who.int/iris/rest/bitstreams/1151838/retrieve.

[2] Ferreira-Borges C., Dias S., Babor T., Esser M.B., Parry CD H. (2015). Alcohol and public health in Africa: Can we prevent alcohol-related harm from increasing?. Addiction.

[3] Morojele N.K., Dumbili E.W., Obot I.S., Parry C. (2021). Alcohol consumption, harms and policy developments in sub-Saharan Africa: The case for stronger national and regional responses. Drug Alcohol Rev..

[4] Jernigan D.H., Trangenstein P.J. (2020). What’s next for WHO’s global strategy to reduce the harmful use of alcohol?. Bull. World Health Organ..

[5] Global Burden of Disease 2016 Alcohol Collaborators (2018). Alcohol use and burden for 195 countries and territories, 1990–2016: A systematic analysis for the Global Burden of Disease Study 2016. Lancet.

[6] Francis J.M., Cook S., Morojele N.K., Swahn M.H. (2020). Rarity and limited geographical coverage of individual level alcohol interventions in sub Saharan Africa: Findings from a scoping review. J. Subst. Use.

[7] Balenger A., Umenze F., Dumbili E., Sako B., Obot I., Swahn M.H. (2021). Developing an alcohol harm prevention research agenda in West Africa: A mixed methods approach. Health Promot. Int..

[8] Swahn M.H., Palmier J.B., May A., Dai D., Braunstein S., Kasirye R. (2022). Features of alcohol advertisements across five urban slums in Kampala, Uganda: Pilot testing a container-based approach. BMC Public Health.

[9] Swahn M.H., Robow Z., Umenze F., Balenger A., Dumbili E.W., Obot I. (2022). A readiness assessment for the prevention of alcohol-related harm in West Africa: A new methodological approach to inform practice and policy. Int. J. Drug Policy.

[10] Movendi International (2019). Kenya Alcohol Law Works. https://cdn.who.int/media/docs/default-source/documents/child-maltreatment/rap-cm-handbook.pdf?sfvrsn=9282691e_2.

[11] Ferreira-Borges C., Esser M.B., Dias S., Babor T., Parry C.D. (2015). Alcohol Control Policies in 46 African Countries: Opportunities for Improvement. Alcohol Alcohol..

[12] Ministry of Health, Republic of Uganda National Alcohol Control Policy. https://www.health.go.ug/cause/national-alcohol-control-policy/.

[13] World Health Organization (2013). Handbook for the Readiness Assessment for the Prevention of Child Maltreatment (RAP-CM). https://www.who.int/violence_injury_prevention/violence/child/handbook_rap_cmp.pdf?ua=1.

[14] Al Eissa M., Saleheen H.N., Almuneef M., Al Saadoon M., Alkhawari M., Almidfa A., Almahroos F. (2019). Child maltreatment prevention readiness in Gulf Cooperation Council (GCC) countries. Int. J. Pediatr. Adolesc. Med..

[15] Al Saadoon M., Al Numani A., Saleheen H., Almuneef M., Al-Eissa M. (2020). Child maltreatment prevention readiness assessment in Oman. Sultan Qaboos Univ. Med. J..

[16] Alkhawari M., Ali K., Al-Abdul Razzaq F., Saleheen H.N., Almuneef M., Al-Eissa M.A. (2020). Multidimensional model to assess the readiness of Kuwait to implement evidence-based child maltreatment prevention programs at a national level. Public Health.

[17] Almuneef M.A., Qayad M., Noor I.K., Al-Eissa M.A., Albuhairan F.S., Inam S., Mikton C. (2014). Multidimensional model to assess the readiness of Saudi Arabia to implement evidence based child maltreatment prevention programs at a large scale. Child Abus. Negl..

[18] Shanley J.R., Armistead L.P., Musyimi C., Nyamai D., Ishiekwene M., Mutiso V., Ndetei D. (2021). Engaging community voices to assess Kenya’s strengths and limitations to support a child maltreatment prevention program. Child Abus. Negl..

[19] World Health Organization Technical Report on the Assessment of Readiness to Implement Evidence-Based Child Maltreatment Prevention Programmes of Brazil, the Former Yugoslav Republic of Macedonia, Malaysia, Saudi Arabia, and South Africa. https://www.who.int/docs/default-source/documents/child-maltreatment/rap-cm-readiness-in-5-countries.pdf?sfvrsn=8bb4c5ce_2.

[20] World Health Organization (2018). SAFER: A World Free from Alcohol Related Harms. https://apps.who.int/iris/rest/bitstreams/1261160/retrieve.

[21] World Health Organization Readiness Assessment for the Prevention of Child Maltreatment: RAP-CM (Short Version—Scoring System). https://www.who.int/docs/default-source/documents/child-maltreatment/rap-cm-short-questionnaire-scoring-sheet.docx?sfvrsn=3103bcca_2.

[22] Ezeanolue E.E., Menson W., Patel D., Aarons G., Olutola A., Obiefune M., Dakum P., Okonkwo P., Gobir B., Akinmurele T. (2018). Gaps and strategies in developing health research capacity: Experience from the Nigeria Implementation Science Alliance. Health Res. Policy Syst..

[23] Sam-Agudu N.A., Paintsil E., Aliyu M.H., Kwara A., Ogunsola F., Afrane Y.A., Onoka C., Awandare G.A., Amponsah G., Cornelius L.J. (2016). Building sustainable local capacity for global health research in West Africa. Ann. Glob. Health.

[24] Defor S., Kwamie A., Agyepong I.A. (2017). Understanding the state of health policy and systems research in West Africa and capacity strengthening needs: Scoping of peer-reviewed publications trends and patterns 1990–2015. Health Res. Policy Syst..

[25] Swahn M.H., Culbreth R., Salazar L.F., Kasirye R., Seeley J. (2016). Prevalence of HIV and Associated Risks of Sex Work among Youth in the Slums of Kampala. AIDS Res. Treat..

[26] Sargent J.D., Babor T.F. (2020). The Relationship Between Exposure to Alcohol Marketing and Underage Drinking Is Causal. J. Stud. Alcohol Drugs Suppl..

[27] Parry C.D. (2014). Testing the National Alcohol Policy Score Card (NAPSC) to Assess Progress in Implementing a Comprehensive Policy response to Reduce the Harmful Use of Alcohol in South Africa. Int. J. Alcohol Drug Res..

